# Management of Undeflatable Coronary Balloons During Percutaneous Coronary Intervention: A Case-Based Review and Practical Bailout Algorithm

**DOI:** 10.31083/RCM51754

**Published:** 2026-07-22

**Authors:** Allam Harfoush, Tammam Harfouch, Muntasir Abo-Al-Hayja, Khaled Albouaini, Ammar Ezeldin, Hanady Hamdallah, Abdalazeem Ibrahem

**Affiliations:** ^1^Cardiology Department, North Bristol NHS Trust, BS10 5NB Bristol, UK; ^2^The Faculty of Medicine and Life Sciences, University of Chester, CH1 4BJ Chester, UK; ^3^Cardiothoracic Surgery, Radiology, and Cardiac Imaging Department, Saint Spiridon County Hospital, 700111 Iasi, Romania; ^4^Cardiology Department, Royal Liverpool University Hospital, L7 8YE Liverpool, UK; ^5^Cardiology Department, Liverpool Heart and Chest NHS Foundation Trust, L14 3PE Liverpool, UK; ^6^General Medicinem, Queen Elizabeth Hospital, NE9 6SX Newcastle Upon Tyne, Gateshead, UK; ^7^Department of Primary Care & Population Health, University College London, WC1E 6BT London, UK; ^8^School of Life and Medical Sciences, University of Hertfordshire, AL10 9EU Hertfordshire, UK

**Keywords:** percutaneous coronary intervention, balloon angioplasty, equipment failure, device removal, coronary artery disease, algorithms, cardiac catheterisation

## Abstract

**Background:**

Undeflatable coronary balloons during percutaneous coronary intervention (PCI) are rare but particularly hazardous because persistent intracoronary inflation can cause abrupt coronary occlusion, ischaemia, and haemodynamic collapse. We aimed to synthesise the published experience to define mechanistic patterns, identify recurring predisposing factors, and propose a practical bailout algorithm.

**Methods and Results:**

We performed a case-based review of PubMed, the Cochrane Library, and EBSCO from inception to February 2026. Eligible reports described balloons that could not be deflated while positioned in a coronary artery. A total of 15 coronary cases (2011–2025) met the inclusion criteria. Events occurred most often during post-dilatation or after stent deployment in complex anatomy, frequently involving severe calcification, aorto-ostial segments, chronic total occlusion-like lesions, and settings with limited guide engagement. Mechanistic patterns clustered into (i) intact-system non-deflation, where restoration of an exit pathway (system checks, sustained negative suction, and contrast dilution), followed by controlled decompression (wire puncture or mechanical- or energy-assisted disruption), was feasible; (ii) shaft-compromised events (fracture or detachment), where management shifted from decompression to mechanical retrieval, with surgery reserved for percutaneous failure.

**Conclusion:**

Undeflatable coronary balloons represent distinct failure modes with different viable rescue options. Outcomes appear to be driven by rapid restoration of perfusion and early recognition of shaft integrity. A structured escalation approach prioritising stabilisation, low-risk troubleshooting, controlled decompression, and decisive transition to retrieval, exclusion, or surgery when decompression is impossible could reduce secondary iatrogenic harm.

## 1. Introduction

Percutaneous coronary intervention (PCI) is performed worldwide with high procedural success and low complication rates [1]; nonetheless, device-related failures remain clinically important because they can be abrupt, unpredictable, and difficult to manage in real time [2]. Among these, an undeflatable balloon is uniquely hazardous because persistent inflation may produce immediate coronary occlusion [3]. In contrast to retained guidewire fragments or embolised devices that may allow preserved flow, a balloon that remains expanded inside a coronary artery can rapidly cause severe ischemia, ventricular arrhythmias, hemodynamic collapse, and death if reperfusion is not restored immediately [4]. Complications described in published reports include coronary perforation and tamponade during salvage attempts, with a potential need for emergent surgical rescue [5].

**Fig. 1.**
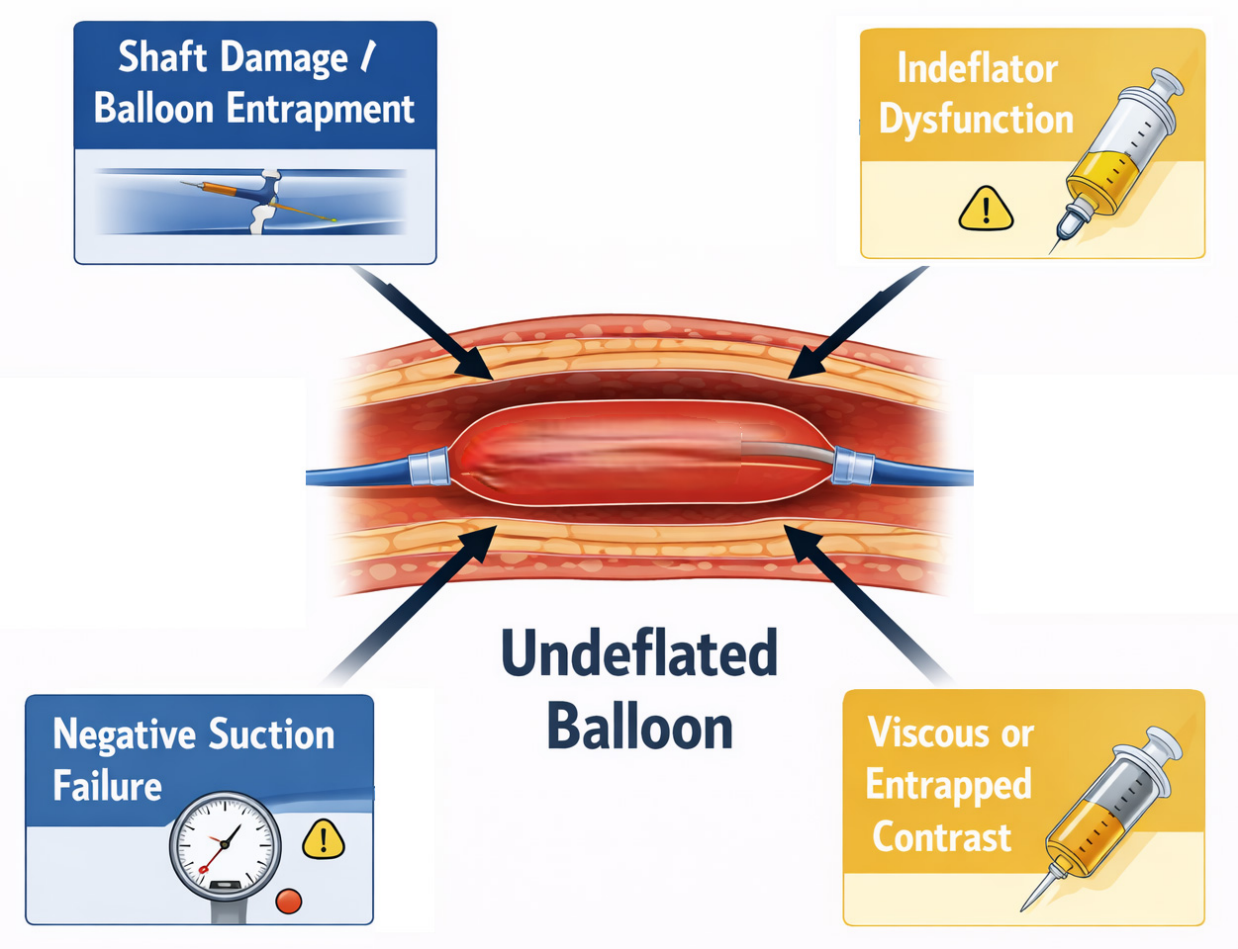
**Summary of the potential balloon deflation failure**.

Because the incidence is extremely low, the literature consists predominantly of case reports and small series with heterogeneous techniques. This case-based review synthesises the retrieved published case reports to provide a focused framework for recognising mechanisms, anticipating risk factors, and selecting bailout strategies, culminating in a practical stepwise management algorithm. Fig. 1 represents a summary of the undeflated balloon.

## 2. Methods

### 2.1 Search Strategy

A literature search was undertaken across three databases, PubMed, Cochrane Library, and EBSCO, from inception till February 2026. The search strategy included the following keywords (PCI OR PTCA OR percutaneous coronary OR coronary angioplasty OR coronary intervention) AND (balloon OR stent balloon OR angioplasty balloon) AND (nondeflat OR non-deflat OR undeflat OR failed to deflate OR failure to deflate OR unable to deflate OR cannot deflate OR entrap OR balloon entrap OR balloon stuck OR retained balloon OR trapped balloon). No language restrictions or additional database filters were applied at the initial search stage. Manual screening of the reference lists of the included cases was also used to identify any further relevant studies.

### 2.2 Selection Criteria

We set of inclusion and exclusion criteria. Inclusion criteria were peer-reviewed publications reporting human cases of an angioplasty balloon, semi-compliant balloon, non-compliant balloon, stent-delivery balloon, cutting balloon, or intravascular lithotripsy (IVL) balloon that could not be deflated (or not be withdrawn due to persistent inflation/entrapment) while positioned in a coronary artery. Exclusion criteria were non-coronary cases (peripheral, neurovascular, structural heart, or non-PCI contexts) and retained coronary hardware where the balloon was not inflated at the time of failure (e.g., isolated guidewire, stent, or catheter retention without deflation failure).

### 2.3 Screening and Study Selection

All identified records were screened by title and abstract to assess eligibility. Where the title/abstract did not provide sufficient detail to determine whether a case met inclusion criteria (e.g., unclear whether the balloon remained inflated or whether entrapment was primarily mechanical), the full text was reviewed. Duplicates were removed by cross-referencing first author, year, journal, procedural scenario, and key case descriptors (target vessel/lesion and failure description). The selection process was structured in line with Preferred Reporting Items for Systematic Reviews and Meta-Analyses (PRISMA) principles (identification, screening, eligibility, inclusion), adapted pragmatically for a case-based narrative review.

### 2.4 Data Extraction and Data Items

Data were extracted using a structured spreadsheet designed a priori for this review. Extracted variables were grouped into patient and clinical context, lesion and procedural characteristics, failure mode classification, bailout management, outcomes, and complications.

### 2.5 Approach to Synthesis and Rationale for Narrative Methodology

A quantitative meta-analysis was not appropriate for this review because the available evidence consisted almost exclusively of single-patient case reports with substantial heterogeneity in failure mechanisms, clinical jeopardy, devices, procedural context, and outcome reporting. Accordingly, we undertook a structured narrative synthesis focused on pattern recognition. This synthesis informed the development of a bailout algorithm, as seen in the next sections.

## 3. Results

**Fig. 2.**
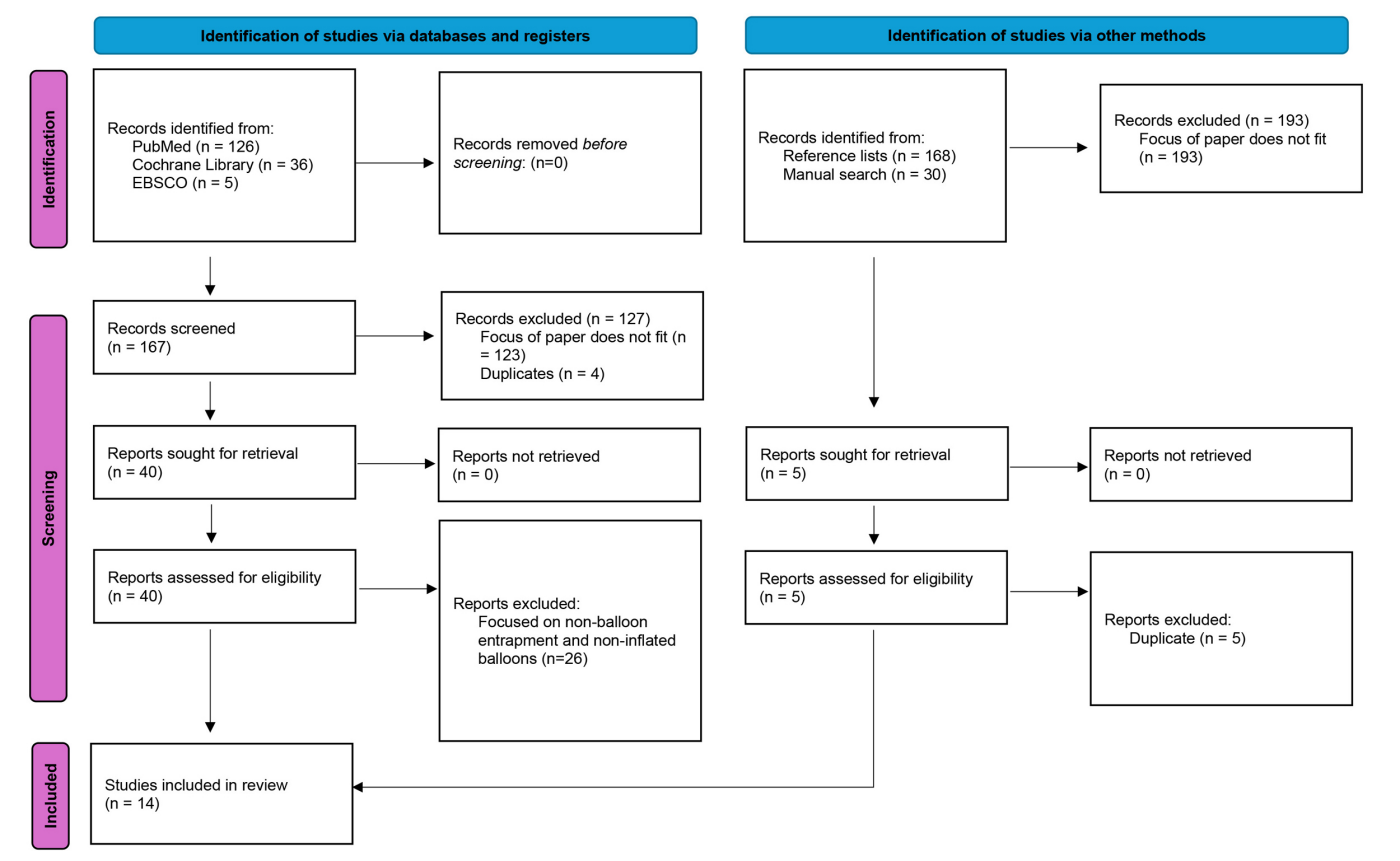
**The PRISMA flowchart of this scoping review**.

Fifteen published case reports/case-based publications describing coronary balloon nondeflation or entrapment during PCI, published between 2011 and 2025, were included (Fig. 2, PRISMA flowchart). Table 1 (Ref. [6,7,8,9,10,11,12,13,14,15,16,17,18,19,20]) summarises case characteristics, suspected failure mechanisms, bailout attempts, definitive management strategies, and outcomes.

**Table 1.** **Outcomes of the undeflatable coronary balloons**.

Mechanism group	First author Year	Vessel	Failure mode	Key bailout attempts	Definitive strategy	Outcome
Wire puncture	Trivedi 2019 [6]	RCA	Suspected lumen collapse	Two deflators change; dye–saline exchange	Controlled wire puncture	Discharged
	Bostan 2013 [7]	LCx	Undeflatable stent balloon	High-pressure burst failed	Deep intubation + puncture	Discharged
	Girish 2011 [8]	LAD	Kink/defect; lumen obstruction	Dilution/aspiration	Deep intubation + puncture	Stable
Shaft-compromised – fracture/detachment	Han 2026 [9]	LM/LAD	Entrapment + shaft detachment	Deflator replacement	Guide-extension en bloc retrieval	Recovered
	Omer 2020 [10]	LM/LAD	Balloon detached	High-pressure inflation	Crush under stents	Uneventful
	Girish 2011 [11]	LCx	Shaft break	Balloon-in-guide trap failed	Modified Fogarty traction	PCI completed
Mechanical perforation (cut-tip technique)	Takama 2015 [12]	LAD	Defective balloon post-dilation	Deflation attempts; shaft transection	Cut-tip anchoring to perforate/burst balloon	Follow-up not reported
High-pressure rupture	Kim 2025 [13]	LAD	Non-deflation; kink	Wire puncture failed	Ultra-high-pressure rupture	Perforations; covered stents
	Yumul 2022 [14]	RCA	Non-deflation	Wire puncture failed	Ultra-high-pressure burst + snare	Successful
Laser-assisted decompression	Savvoulidis 2021 [15]	RCA	Shaft kink/twist	CTO-wire puncture + electrocautery failed	Excimer laser perforation	Uneventful
Traction-based bailout	Chitturi 2025 [16]	RCA	IVL balloon stuck behind calcium	Parallel wire; snare; guide-extension	Shaft severed; manual extraction	PCI completed
	Gibson 2025 [17]	RCA	Undeflatable SC balloon	Wire puncture failed	Inflated balloon + sheath removed	Retroperitoneal haematoma
	Yang 2023 [18]	RCA	One-way valve; failed deflation	Deflator replacement	Withdrawal of inflated balloon into aorta; removed with guide	No injury on IVUS; well at 6 months
	Leibundgut 2018 [19]	RCA	Contrast trapped; hypotube damage	Aortic burst attempt failed; puncture failed	23G transcutaneous needle puncture + aspiration	No radial injury; stable
Surgical management	Okabe 2025 [20]	LAD	NC balloon failed; poor guide engagement	Catheter-based bailout failed	Mini-thoracotomy + direct puncture	Survived

RCA, right coronary artery; LCx, left circumflex artery; LAD, left anterior descending; LM, left main; IVL, intravascular lithotripsy; SC, semi-compliant; NC, non-compliant; CTO, chronic total occlusion; PCI, percutaneous coronary intervention; IVUS, intravascular ultrasound.

Across the included reports, failure to deflate or remove most often occurred during post-dilation or after stent deployment in complex anatomy, including severe coronary calcification, aorto-ostial lesions, chronic total occlusion-like segments, left main interventions, and cases complicated by limited guide engagement, such as post-transcatheter aortic valve implantation (TAVI) access constraints [10,13,15,20]. A modern device-specific variant involved intravascular lithotripsy balloon entrapment behind a calcium shelf despite prior atherectomy, in which conventional retrieval strategies were limited by shaft bending [16].

Definitive management strategies are grouped into several patterns. First, when the system remained intact, operators attempted restoration of the deflation pathway through system checks, sustained negative suction, and contrast dilution with saline, followed by controlled balloon decompression using wire puncture or mechanical perforation techniques. Second, when puncture attempts failed, energy-assisted balloon disruption with excimer laser was successfully used to facilitate decompression and retrieval [15]. Third, when the shaft fractured or the balloon detached, the strategy shifted from decompression to mechanical salvage. Guide-extension–assisted anchoring and en bloc retrieval was successful during left main PCI complicated by cardiac arrest [9], while intentional exclusion by crushing the detached inflated balloon under stents was used to re-establish flow in a left main bifurcation case following TAVI [10]. Finally, when catheter-based solutions failed or were technically infeasible, surgical rescue was described, including a minimally invasive approach using small thoracotomy and direct needle puncture of the balloon without coronary incision [20]. A high-risk last-resort strategy, intentional rupture of the balloon at ultra-high pressure within the coronary bed, enabled retrieval but has the risk of coronary perforations requiring covered stents [13].

### 3.1 Mechanistic Patterns and Why They Matter Clinically

The case literature demonstrates that an “undeflatable balloon” represents a set of distinct failure mechanisms rather than a single entity. This distinction is clinically important because it determines which salvage strategies remain viable. When the balloon shaft and hypotube remain intact, the operator can attempt to re-establish an exit pathway for contrast using sustained negative suction, contrast dilution, and system troubleshooting before progressing to controlled decompression techniques such as wire puncture or mechanical perforation. In contrast, once the shaft fractures or the balloon detaches, classic decompression through the balloon catheter becomes impossible, and management shifts to mechanical salvage.

A useful critical lens is to separate “true non-deflation” from “delayed deflation”. Bench data show that balloon emptying is strongly affected by inflation-media viscosity and dilution: lower-viscosity contrast and greater saline dilution significantly shorten deflation times [21]. This is clinically relevant because mislabelling a viscosity as “device failure” can prompt premature escalation (e.g., puncture or traction), increasing the risk of dissection and perforation. In other words, mechanism classification is a safety step, as it reduces avoidable escalation when time and physiology allow [3].

Conversely, once hydraulic continuity is lost, troubleshooting becomes low-yield and time-consuming because the balloon can no longer vent through its designed pathway. At that point, the event aligns with the “entrapped gear” framework where priority shifts to restoring flow and minimising vessel trauma through structured retrieval options [22].

Finally, it is worth highlighting that “deflation behaviour” is treated as a device performance characteristic, not an afterthought. International standards for balloon dilatation catheters include specific testing provisions for balloon deflation time [23], and deviation from expected behaviour should trigger a structured response rather than forceful manipulation.

### 3.2 Decompression Techniques (Puncture, Perforation, and Energy-Assisted Disruption)

Wire puncture is conceptually attractive because it aims to create a small, predictable vent. In practice, several technical variants of balloon puncture have been described. A controlled method involves advancing a parallel guidewire through a microcatheter positioned at the proximal balloon shoulder, a concept analogous to the pierced‑balloon technique used for controlled membrane perforation in complex bifurcation wiring [24]. The microcatheter provides axial stability and precise targeting, allowing the operator to expose only 1–2 mm of the wire tip. A high‑penetration wire or even the stiff back end of a standard 0.014″ coronary wire can be used to create a focal perforation in the balloon wall, as demonstrated in published reports of nondeflating stent balloons [11]. Once the microcatheter is stabilised, gentle advancement of the wire produces a sudden loss of resistance indicating balloon entry, followed by aspiration through the balloon catheter.

When mechanical puncture is unsuccessful, energy-assisted disruption (excimer laser) is a potential adjunct when mechanical puncture fails or is not feasible, but it should be framed as a high-resource option with its own perforation-risk profile. Registry-era data for excimer laser coronary angioplasty show that perforation can occur and is associated with worse downstream outcomes [25]. Practically, if Excimer is used, it should be explicitly projected as a bailout strategy rather than a routine approach [26].

### 3.3 High‑Risk Manoeuvres: Traction and Intentional Rupture

Forceful traction and intentional balloon rupture represent high-risk manoeuvres that can convert a potentially manageable non-deflation event into a retained-device or perforation catastrophe [27]. Forceful traction can escalate the problem. Excessive pulling may convert a potentially manageable nondeflation scenario into a retained-device catastrophe by causing hypotube disruption, shaft fracture, or complete balloon detachment, thereby prolonging ischemic time and limiting available rescue options. Some operators describe withdrawing the inflated system into the aorta to re-establish coronary flow when other manoeuvres fail, but this should remain exceptional because the same traction that restores flow can also produce ostial/segmental dissection or stent deformation, with consequences that may be angiographically subtle at first [28].

Intracoronary rupture carries a recognised risk of perforation requiring covered stents or surgery [29]. This is not just theoretical, as data show that perforation, while uncommon, carries clinically meaningful risk (covered stents were necessary in ~25% and emergency surgery in ~17%) [30]. If either manoeuvre is used as a last resort, immediate angiographic reassessment is essential to exclude dissection, stent deformation, or thrombosis [31].

### 3.4 When Percutaneous Options Fail

Surgical rescue remains definitive in two situations: 1—when the retained device cannot be safely retrieved percutaneously, and 2—when catheter manipulation risks further harm. The Society for Cardiovascular Angiography & Interventions (SCAI) expert consensus on PCI systems and emergency surgical support is explicit that emergency surgery may be required for retained devices that cannot be managed with percutaneous approaches. Clinically, this supports a low threshold for early Heart Team activation once repeated percutaneous attempts become low yield [32]. This threshold may be even lower in anatomically constrained settings such as post-TAVI coronary re-engagement, where guide engagement and coaxial support can be limited. Reviews and observational work on coronary access after TAVI document that re-access feasibility varies by valve design and implantation geometry, limiting procedural control and rescue capability [33]. Notably, newer transcatheter valve platforms have incorporated commissural alignment strategies and larger/wider stent cells designed to improve coronary re-engagement, which may reduce, but not eliminate, access limitations during subsequent PCI [34].

## 4. Predisposing Factors of Deflation Failure

Lesion anatomy appears to play a central role. Severe calcification and acute recoil, particularly in ostial disease, may compress the balloon’s entry/exit port, trap contrast, and mechanically constrain the balloon within rigid calcium. Shelf-like calcium capable of physically locking devices is implicated in IVL entrapment despite prior lesion modification. Proximal large-vessel involvement (left main, proximal left anterior descending (LAD), ostial right coronary artery (RCA)) increases the consequences of even brief occlusion and may necessitate early hemodynamic support and early escalation to definitive rescue options.

Procedural mechanics also contribute. Kinking, twisting, stretching, or lumen collapse of the hypotube/shaft during delivery or pullback can prevent negative suction from evacuating contrast or create a one-way valve effect that maintains balloon inflation. Importantly, forceful withdrawal against resistance can cause shaft fracture or balloon detachment, transforming a decompression problem into a retained-device problem with fewer options. Device/system factors, including suspected manufacturing defects, are raised when abnormal inflation/deflation behavior is present from the outset and no obvious mechanical explanation is identified. Finally, system-level constraints, such as poor guide engagement after TAVI, may preclude effective guide-extension use and reduce catheter-based feasibility, accelerating the need for surgical consultation. A summary of the observed factors across the reports is presented in Table 2.

**Table 2.** **Predictors of balloon deflation failure or entrapment in PCI**.

Group	Factors	What it predicts
Lesion/anatomy	Severe calcification	Higher risk of non-deflation
	Ostial lesion	Higher risk of non-deflation
	Calcium ledge/shelf	Higher risk of entrapment
	Marked lesion recoil	Higher risk of non-deflation
Device/system	Resistance during delivery	Risk of kink/shaft damage and non-deflation
	Shaft/hypotube kinking, twisting, or lumen collapse	Higher risk of non-deflation
	Resistance during withdrawal	Higher risk of fracture/detachment
	Abnormal inflation/deflation behaviour first use	Suggests an intrinsic device malfunction
	Limited guide support/engagement	Lower likelihood of catheter-based rescue success

## 5. Proposed Management Algorithm

**Fig. 3.**
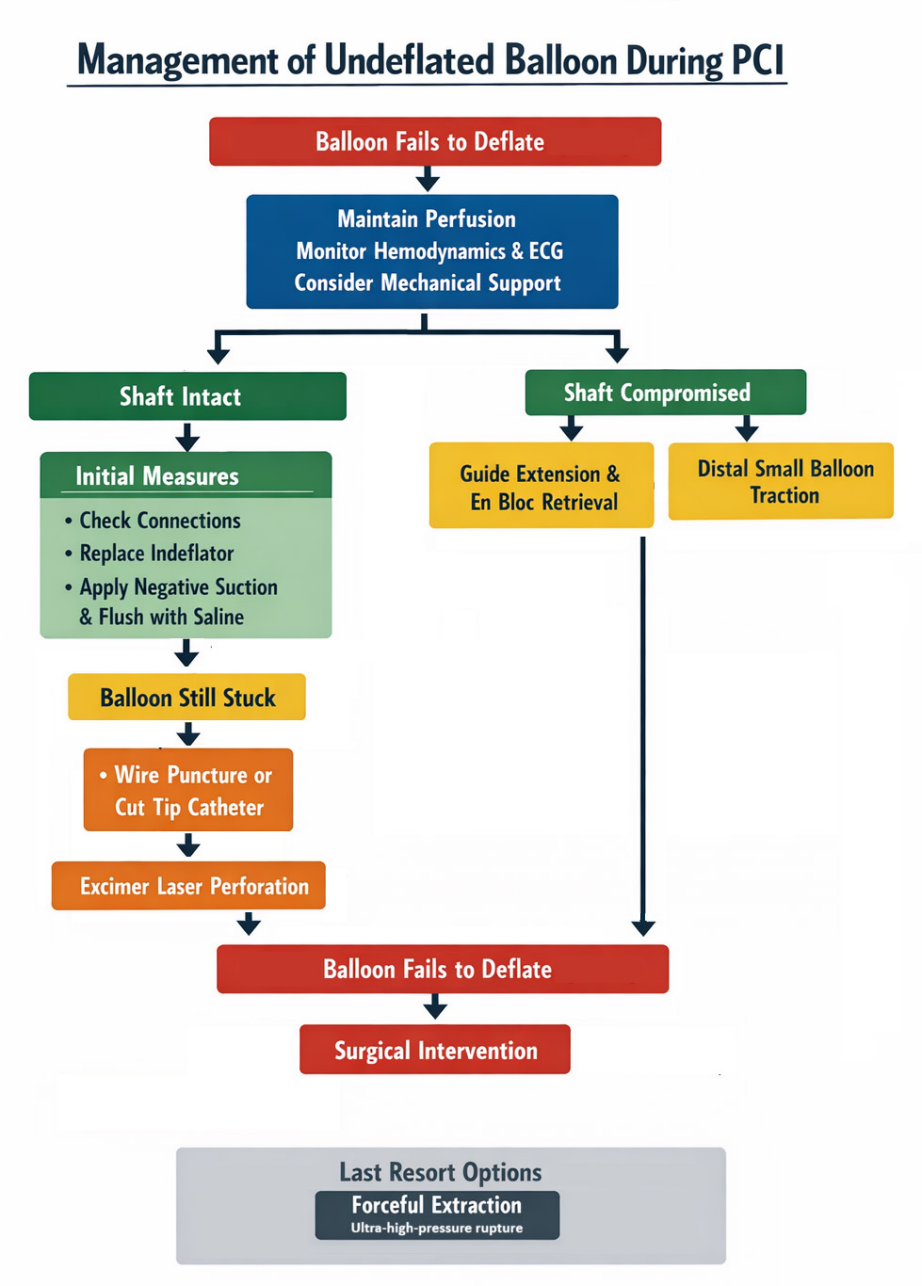
**The suggested algorithm for the management of non-deflating balloons**. ECG, electrocardiogram.

The proposed approach is principle‑led rather than step‑led (Fig. 3). The first objective is time‑sensitive myocardial protection: maintain coronary perfusion and anticipate deterioration with continuous electrocardiogram (ECG) and hemodynamic surveillance, with a low threshold for mechanical circulatory support in high‑risk territories.

The second objective is to preserve rescue optionality. If the system appears intact, initial management should prioritise low‑risk troubleshooting: verify stopcock connections, replace the indeflator, apply sustained negative suction, and dilute contrast with saline.

Escalation is guided by a single decision point: whether hydraulic continuity is preserved.

If continuity is preserved, controlled decompression techniques such as wire puncture or, in experienced centres, excimer‑laser–assisted disruption may be used.

If shaft integrity is lost, management shifts to mechanical retrieval, typically using guide‑extension anchoring and en bloc extraction.

If percutaneous options fail or are not feasible, early surgical consultation is appropriate.

Intentional rupture and forceful traction should be reserved for exceptional circumstances only, with immediate post‑manoeuvre angiographic reassessment.

In practice, decision‑making depends on the patient’s hemodynamic status, the coronary territory at risk, the integrity of the balloon shaft and hypotube, the degree of mechanical entrapment, and the availability of guide‑extension catheters, excimer laser systems, and surgical backup.

## 6. Conclusions

Undeflatable coronary balloons are rare but potentially fatal complications of PCI. The published experience indicates that outcomes are determined primarily by the underlying failure mode and by how quickly coronary perfusion is restored in high-jeopardy territories. A structured escalation approach should prioritise early stabilisation, low-risk troubleshooting, controlled decompression, and early transition to mechanical retrieval/surgery when decompression becomes impossible, which would reduce secondary harm.
